# Prognostic Factors and Life Expectancy in Canine Leishmaniosis

**DOI:** 10.3390/vetsci7030128

**Published:** 2020-09-04

**Authors:** Maria Aires Pereira, Rute Santos, Ricardo Oliveira, Lina Costa, Ana Prata, Vânia Gonçalves, Madalena Roquette, Helena Vala, Gabriela Santos-Gomes

**Affiliations:** 1 Global Health and Tropical Medicine, GHTM, Instituto de Higiene e Medicina Tropical, IHMT, Universidade Nova de Lisboa, UNL, R. da Junqueira 100, 1349-008 Lisbon, Portugal; santosgomes@ihmt.unl.pt; 2 Agrarian School of the Polytechnic Institute of Viseu, Quinta da Alagoa-Estrada de Nelas Ranhados, 3500-606 Viseu, Portugal; hvala@esav.ipv.pt; 3 Polytechnic Institute of Portalegre, Praça do Município 11, 7300-110 Portalegre, Portugal; rutesantos@ipportalegre.pt (R.S.); ricardooliveira@ipportalegre.pt (R.O.); lina_costa@ipportalegre.pt (L.C.); madaroquett@gmail.com (M.R.); 4 VALORIZA—Research Centre for Endogenous Resource Valorization, Campus Politécnico, 10, 7300-555 Portalegre, Portugal; 5 Clilegre-Clínica Veterinária de Portalegre, Rua Martinho Azevedo Coutinho nº 13A e 16A, 7300-817 Portalegre, Portugal; 6 Vetviana-Consultório Veterinário, Rua Padre Luís António da Cruz 67, 7090-284 Viana do Alentejo, Évora, Portugal; anamprata7@hotmail.com; 7 Centro Veterinário da Vidigueira, Largo Frei António das Chagas 25A, 7960-220 Vidigueira, Beja, Portugal; centrovetvidigueira@gmail.com; 8 VetAlter-Clínica Veterinária, Avenida Padre José Agostinho Rodrigues nº 13, 7440 Alter do Chão, Portalegre, Portugal; 9 Centre for the Research and Technology of Agro-Environmental and Biological Sciences (CITAB), University of Trás-os-Montes and Alto Douro, 5001-801 Vila Real, Portugal

**Keywords:** canine leishmaniosis, prognostic factors, survival time, IRIS staging, therapy selection and management

## Abstract

Canine leishmaniosis (CanL) is a chronic and potentially fatal disease. The prognosis of CanL depends on the severity of the clinical signs and clinicopathological abnormalities presented by the dog at the time of diagnosis. This study aims to estimate the survival time of dogs with CanL, determining the prognostic value of different clinical and clinicopathological parameters. Medical records of 99 dogs diagnosed with CanL in five veterinary centers of the Alentejo region (Portugal) were examined retrospectively. The majority of dogs presented hyperproteinemia, moderate normocytic normochromic anemia, normal blood urea and creatinine levels and were classified as stage 1 according to the International Interest Society (IRIS) guidelines at the time of diagnosis. The severity of anemia, presence of concomitant infectious diseases at the time of diagnosis and the anti-*Leishmania* therapy were correlated with the survival time. The influence of renal dysfunction was evaluated by Receiver Operating Characteristic (ROC) curve and survival analysis. Survival analysis demonstrated that patients classified as IRIS 1 at the time of diagnosis survived more than four years, in contrast with dogs classified as IRIS 2 that survived around two and half years and dogs classified as IRIS 3–4 that survived around one month. IRIS stage deteriorated during the course of CanL in one third of the dogs and was the principal cause of death or euthanasia in a high proportion of animals. In some cases, dogs did not receive anti-*Leishmania* treatment or abandoned the veterinary follow-ups, which may have considerable repercussions for animal wellbeing and public health. This study reinforces the value of blood urea and creatinine levels as prognostic factors in CanL.

## 1. Introduction

Canine leishmaniosis (CanL) is a zoonosis mainly caused by the parasite *Leishmania infantum*, which is transmitted by phlebotomine (sand flies). CanL is endemic in more than 70 countries from South and Central America, the Mediterranean region, Africa and Asia [1,2,3]. Seroprevalence of *L. infantum* infection varies from 5% to 30% in the Mediterranean basin [3], estimating that at least 2.5 million dogs are infected [4]. Furthermore, CanL is spreading northward [5,6,7,8], and it is considered emergent in non-endemic countries, such as Germany [9], the Netherlands [10] and Poland [11], due to dog importation and traveling [12].

*L. infantum* infection can manifest as subclinical, self-limiting illness or as a severe and life-threatening disease, generally presenting chronic evolution [3,13]. Common clinical signs include skin lesions, weight loss, lymphadenopathy [14,15,16] and splenomegaly [13]. Bone marrow involvement explains, in part, some clinicopathological abnormalities related to CanL, as is the case of anemia and thrombocytopenia [16,17]. The kidney is affected in virtually all dogs [18], but azotemia is an infrequent clinicopathological finding [2]. Renal disease can progress from asymptomatic proteinuria to nephrotic syndrome and/or chronic kidney disease (CKD) [3,19,20]. Ocular disease, bleeding disorders and arthritis can also be observed, as well as, less frequently, digestive and neurologic diseases [3,16].

The prognosis of CanL, which is the probability of a patient developing a particular outcome over a specific period [21], seems to depend on the severity of the clinical signs and clinicopathological abnormalities, especially the degree of renal dysfunction at the time of diagnosis [13,22,23]. International Renal Interest Society (IRIS) guidelines [24] assist the clinical classification of CanL according to disease severity [3] and are used to follow the response to anti-*Leishmania* treatment and for prognosis [25,26]. IRIS guidelines establish four stages of CKD based on blood creatinine levels and three sub-stages based on proteinuria (urine protein to creatinine ratio, UP/C) assessed on at least two occasions in a hydrated, stable patient. These guidelines provide practicing veterinarians with evidence-based guidance for diagnosing, treating and managing kidney disease [24]. 

Proteinuria, hypoalbuminemia and lymphopenia have been correlated with short survival of treated dogs living in non-endemic areas [22]. However, so far, few studies have specifically addressed this issue. Coinfections with other pathogens are frequently reported in dogs with CanL [27,28,29]. Some of these infectious diseases mimic the clinical signs and/or clinicopathological abnormalities of CanL [29,30,31,32], affect the severity of CanL [29,31,32,33] and can influence its prognosis. 

Therapy with anti-*Leishmania* drugs promotes the clinical cure, increases life expectancy [22,34,35,36] and decreases canine infectiousness to sand fly vector [35,37,38,39,40,41,42]. Meglumine antimoniate in combination with allopurinol (Me + A) is considered the “gold standard” treatment in the absence of severe clinical conditions [43,44], whereas miltefosine plus allopurinol (M + A) or allopurinol alone (A) are alternative treatments that are available for oral administration [45,46]. Second choice drugs include aminosidine (Am), amphotericin B deoxycholate and immunomodulator-based treatments [35,44,47]. However, despite adequate treatment, dogs rarely achieve the parasitological cure and eventually relapse [48,49]. Relapses are associated with increased clinical scores [49], which can increase the infectivity of dogs to sand fly vectors, since it was shown that symptomatic dogs have a higher probability of infecting the vector [42,50].

Therefore, ongoing patient assessment is crucial for the early identification and treatment of relapses. Monitoring CanL patients requires periodical and long-term assessment of the possible toxic effects of the treatment, the clinicopathological status (renal function, inflammation/immune response) and the parasitological status [23,51], which comprises a long follow-up period and entails high costs for dogs’ tutors [44].

Thus, this study aims to estimate the survival time of dogs with CanL and examine the prognostic value of different clinicopathological parameters to improve clinical decisions.

## 2. Materials and Methods

### 2.1. Ethical Approval

This retrospective study has been performed on client-owned dogs referred to veterinary centers for therapeutic purpose. All owners gave informed consent for treatment, laboratory tests and for data recording. The study was carried out in accordance with Portuguese law (Decree Law No. 1/2019) and European Union legislation (EU Directive 2010/63) covering the use of animals for scientific purposes.

### 2.2. Case Selection

**Figure 1:**
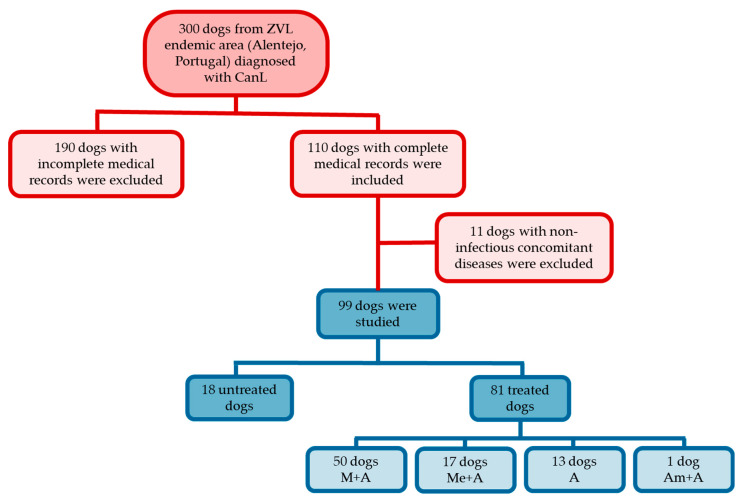
Flowchart representing the case selection process used in the current study. From 300 dogs living in an endemic area of zoonotic visceral leishmaniosis (ZVL) diagnosed with canine leishmaniosis (CanL), 99 dogs were selected and evaluated. Dogs were treated with miltefosine in combination with allopurinol (M + A), meglumine antimoniate in association with allopurinol (Me + A), allopurinol in monotherapy (A) or aminosidine in combination with allopurinol (Am + A).

The medical records of 300 dogs diagnosed with CanL between 1 January 2014 and 31 May 2019 at five veterinary centers located in Alentejo (NUTS III, Portugal) were screened and data extracted. Medical records meeting the following criteria were taken into consideration for the current study: animal signalment, clinical signs and clinicopathological findings (hematology and biochemical profile) exhibited by the dog at the time of diagnosis before starting therapy and during the evolution of the disease, results of specific diagnostic tests for *Leishmania* infection and anti-*Leishmania* treatment prescribed. Clinical records of dogs with clinical signs and/or clinicopathological findings consistent with concurrent neoplastic, inflammatory, endocrine, immunologic and genetic diseases were excluded. The medical records of 99 dogs fulfilled the selection criteria and were studied retrospectively (Figure 1).

### 2.3. Medical Records Evaluation

#### 2.3.1. Dog Signalment

Dog signalment was extracted from the clinical records of each animal. Dogs were categorized according to sex and breed and classified into the following age groups: young (<1 year), young adult (1–3 years), adult (4–8 years) and mature (>8 years). Date of birth and date of CanL diagnosis were used to determine the age of the dog at the time of diagnosis.

#### 2.3.2. Establishment of CanL Diagnosis

CanL diagnosis was confirmed based on the information extracted from the clinical records. Clinical disease (CanL) was confirmed by positive serological tests and/or by detection of *Leishmania* amastigotes in tissue samples combined with clinical signs and/or clinicopathological (hematological, biochemical) abnormalities characteristic of CanL [3,13].

Detection of anti-*Leishmania* antibodies was performed by commercial immunofluorescence antibody test (IFAT), enzyme-linked immunosorbent assay (ELISA) or by rapid immunochromatographic test (RIT). IFAT titers were considered positive above the laboratory cut-off (1:80) and ELISA above 1.1. Levels of antibodies anti-*Leishmania* obtained by IFAT and ELISA were categorized as low (1:160; 1.1–1.5), high (1:320; 1.5–2.9) and very high (>1:320; >2.9). IFAT and ELISA tests were carried out by different commercial laboratories (DNAtech – Investigação Científica e Análises Moleculares, Lisboa, Portugal; Cedivet – Centro Diagnóstico Veterinário, Porto, Portugal; INNO, Serviços Especializados em Veterinária, Braga, Portugal). *Leishmania* amastigotes were detected by direct microscopy in biological samples (skin biopsies, spleen and skin cytology) (INNO – Serviços Especializados em Veterinária).

Compatible clinical signs and clinicopathological abnormalities [14,15,16] presented by dogs at the time of diagnosis were extracted from clinical records. In the majority of cases, hematology and biochemistry were determined by in-house analyzers at each veterinary center. Hematocrit values were used to classify anemia as mild (30–37%), moderate (20–30%) or severe (<20%). Urea values were considered normal between 6 and 24 mg/dL. Blood creatinine levels registered in the clinical records were used to classify patients according to IRIS stage as IRIS 1 (serum creatinine < 1.4 mg/dL), IRIS 2 (1.4–2.8 mg/dL), IRIS 3 (2.9–5 mg/dL) and IRIS 4 (>5 mg/dL) (IRIS) [24] at the time of diagnosis and during the evolution of the disease.

#### 2.3.3. Confirmation of Other Infectious Diseases

*Ehrlichia canis*, *Babesia canis*, *Rickettsia conorii*, *Leptospira* spp. and *Neospora caninum* were ruled out by IFAT (DNAtech– Investigação Científica e Análises Moleculares) whenever there was clinical suspicion. IFAT titers were considered positive above the laboratory cut-off, which was 1:50 for *E. canis*, 1:32 for *B. canis*, 1:40 for *R. conorii*, 1/200 for *Leptospira* spp. IgM and IgG and 1:32 for *N. caninum*. *Thelazia callipaeda* infestation was ruled out by visual inspection.

#### 2.3.4. Anti-*Leishmania* Treatment

Different anti-*Leishmania* therapies were applied to sick dogs, namely M + A, Me + A, A or aminosidine in combination with allopurinol (Am + A). Dogs treated with M + A received miltefosine (Milteforan®, Virbac S.A, France; 2 mg/kg *per os*, *semel in die*—SID—for 4 weeks) in association with allopurinol (Zyloric^®^, Laboratórios Vitória, Portugal; 10 mg/kg, *per os*, *bis in die*—BID—*ad eternum* in the majority of the cases). Dogs treated with Me + A received meglumine antimoniate (Glucantime^®^, Merial Portuguesa, Portugal; 100 mg/kg SID for 4 weeks) in association with allopurinol at the previous dose. A small proportion (6.7%) of dogs treated with combined therapy received a second course of M or Me. A in monotherapy was administered at the dosage indicated above. Finally, the dog treated with Am + A received aminosidine (Gabbrocol^®^, CEVA, Portugal) at the dose of 15 mg/kg, subcutaneously, SID, for 28 days plus allopurinol.

#### 2.3.5. Veterinary Follow-Ups

The date of the last contact with the veterinary center or the date of death (natural or by euthanasia) were extracted from clinical records. Fifty-six dogs were followed until natural death occurred or until they were euthanized at veterinary centers where diagnosis was established, being registered at the time of death. Data collected from these dogs were considered as deaths/events in the survival analysis. Forty-three patients were lost during follow-up or stayed alive and data obtained from these animals were regarded as censored data in the survival analysis.

### 2.4. Statistical Analysis

A survival analysis using the Kaplan–Meier product-limit method was performed to assess inter-group differences in survival times between dogs with and without clinicopathological abnormalities. Survival time was defined by the number of days that passed between diagnosis and death. When it was not possible to obtain the date of death, the last contact with the veterinary center was used as the date of censoring. The influence of different variables on survival time was assessed by the log-rank test. A receiver operating characteristic (ROC) analysis was performed to assess the predictive value [52] of the IRIS value of 1 (normal) and a normal urea value at CanL diagnosis for survival time. The nonparametric Mann–Whitney test was used to compare survival times between treatments (*p* < 0.05). Survival analysis and Mann–Whitney test were performed on Graph Pad Prism 6 and ROC analysis on SPSS Statistics 26.

## 3. Results

### 3.1. Anemia and Hyperproteinemia Are Frequent Clinicopathological Findings in CanL

The majority of sick dogs were mixed breed (47.5%) and Labrador retriever (16.2%), but the disease also was diagnosed in another 10 purebreds. The median age was 5.58 years (range 0.67–14.25 years), with over-representation of adult dogs (53.5%), followed by mature dogs (27.3%) and young adult dogs (18.2%). Fifty-nine dogs (59.6%) were male and 40 (40.4%) female (Table 1).

In 96 dogs (97.0%), the diagnosis was established based on positive serological tests associated with clinical signs and/or clinicopathological abnormalities compatible with CanL, and, in two of these dogs, the parasite was identified in skin biopsy. Three animals presented doubtful serological tests (IFAT 1:80 in two animals; RIT doubtful in one dog); thus, CanL diagnosis was established based on the detection of *Leishmania* amastigotes in the skin (two dogs) and spleen cytology (one dog), associated with clinical signs and/or clinicopathological abnormalities. Clinical signs compatible with CanL were observed in 95 (96.0%) dogs. In the remaining four animals, CanL diagnosis was established based on compatible clinicopathological abnormalities. 

The most prevalent clinical signs observed in CanL patients were dermatological lesions (seborrheic dermatitis, hyperkeratosis, skin ulcers, onychogryphosis) (67.4%), weight loss (40.0%), lymphadenomegaly (21.0%), osteoarticular pathology and muscle atrophy (18.9%), ocular pathology (anterior uveitis, blepharitis, keratoconjunctivitis) (14.7%) and splenomegaly, among others (Table 2).

The most frequent clinicopathological abnormality at the time of diagnosis was anemia (60.4%), with 44.8% of the patients presenting mild normocytic normochromic anemia, 51.7% moderate anemia and 3.4% severe anemia. Total leukocyte count was within the reference range in the majority (82.8%) of the patients, and monocytosis was the most frequent (18.0%) abnormality observed in the leukogram. Thrombocytopenia was detected in 18.3% of the dogs. Hyperproteinemia was detected in 52.8% and hypoproteinemia in 8.8% of the dogs. The hepatic enzymes alkaline phosphatase (ALP) and glutamic-pyruvate-transaminase (GPT) were high in 12.0% and 10.4% of the patients, respectively. Blood urea levels were elevated in 43.4% of the dogs. According to blood creatinine levels, 69.7% of the patients were classified as IRIS 1, 15.1% as IRIS 2, 7.1% as IRIS 3 and 8.1% as IRIS 4 (Table 3).

Detection of anti-*Leishmania* antibodies was performed by IFI (53.5%) and ELISA (26.3%) in the majority of patients. Levels of anti-*Leishmania* antibodies were classified as doubtful in 2, low in 42, high in 18 and very high in 17 dogs. However, in 20.2% of the dogs, serological diagnosis was established by RIT and positive results were not confirmed by quantitative serological tests due to economic restrictions.

The majority of patients (81.8%) received anti-*Leishmania* treatment, of which 61.7% received M + A, 21% Me + A, 16% A in monotherapy and 1.2% Am + A.

### 3.2. Clinicopathological Abnormalities Observed at the Time of Diagnosis Can Be Useful to Predict Prognosis

**Figure 2:**
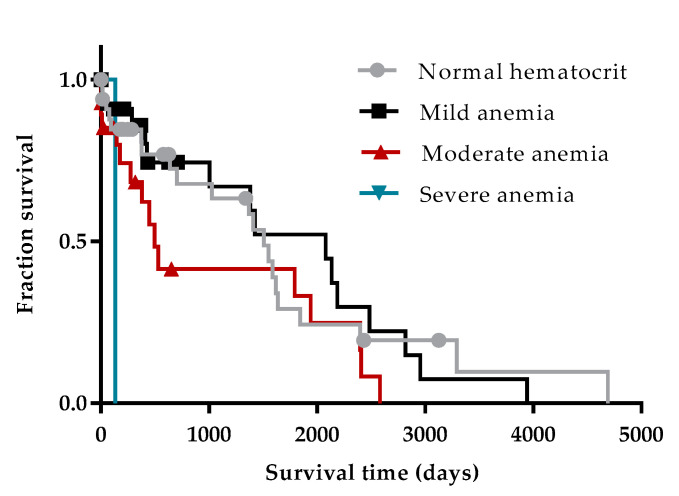
Influence of hematocrit value at the time of diagnosis on the survival time of patients with CanL. Log-rank (Mantel-Cox) test was used to compare the survival time of patients with normal hematocrit values, mild, moderate and severe anemia. Both events (death) and censored data were considered. Dots, triangles and squares represent censored dogs.

The median survival time of patients without (1508 days) and with anemia (1381 days) at the time of diagnosis were not significantly different. The survival time of animals with mild, moderate and severe anemia was 2078, 498 and 136 days, respectively. A significant increase in survival time was observed in patients with mild anemia compared with those with severe anemia (*p* = 0.0180) (Figure 2).

**Figure 3:**
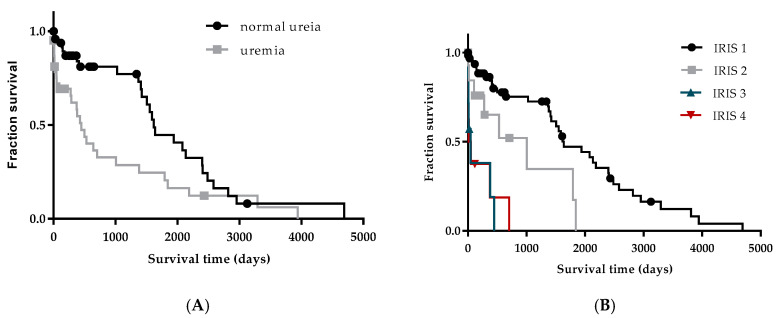
Influence of renal profile at the time of diagnosis on the survival time of patients with CanL. Blood creatinine levels were used to classify CanL patients according to the IRIS stage. Log-rank (Mantel-Cox) test was used to compare the survival time between animals with normal and abnormal blood urea levels (**A**) and between animals within different International Renal Interest Society (IRIS) stages (**B**). Both events (death) and censored data were considered. Dots, triangles and squares represent censored dogs.

The renal profile at the time of diagnosis influenced survival time. The median survival time of dogs with normal blood urea levels (1618 days) was significantly higher than that of dogs with elevated urea levels (448 days) (*p* = 0.0073) (Figure 3A). The median survival time of patients classified as IRIS 1, 2, 3 and 4 was 1637, 1006, 45 and 33.5 days, respectively. Survival time was significantly higher for patients classified as IRIS 1 compared with IRIS 2 (*p* = 0.0113), IRIS 3 and 4 (*p* < 0.0001), for IRIS 2 compared with IRIS 3 (*p* = 0.0128) and IRIS 4 (*p* = 0.0171) and for IRIS 3 compared with IRIS 4 (*p* = 0.01889) (Figure 3B).

**Figure 4:**
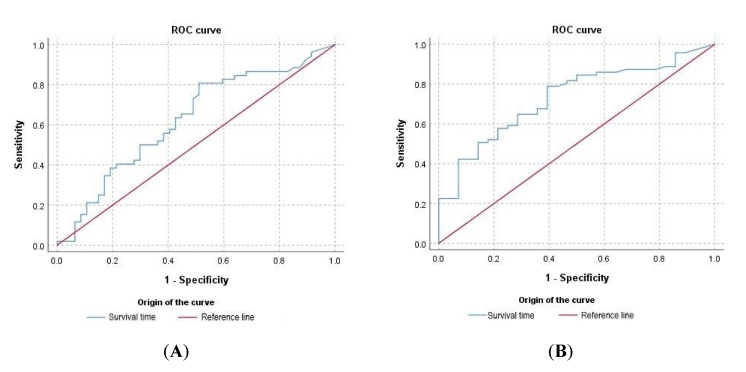
Influence of blood urea levels and IRIS stage on the survival time of patients with CanL. Receiver Operating Characteristic (ROC) curves were used to assess the survival time predictive value of a normal urea value (**A**) or IRIS value of 1 (normal) (**B**) at the time of diagnosis in survival time. Both events (death) and censored data were considered.

The influence of blood urea levels and the IRIS stage on survival time was confirmed by ROC analysis. Normal urea level at the time of diagnosis showed a predictive value for survival time of 62.5% (*p* < 0.05), with an overall model quality of 0.51 (Figure 4A). On the other hand, ROC analysis showed a predictive value of IRIS classification at diagnosis (1 − normal) for survival time of 72.6% (*p* < 0.05), with an overall model quality of 0.62 (Figure 4B).

The influence of renal dysfunction on survival time also was evaluated when only events (deaths) were considered. In this case, 64.3% (*n* = 36/56) of the patients exhibited a normal renal profile and a significantly high median survival time (1508 days) compared with IRIS 2 (531 days, *p* = 0.0469) and IRIS 3 and IRIS 4 (29 and 15 days, respectively, *p* < 0.0001) at the time of diagnosis. ROC analysis revealed that normal urea levels at the time of diagnosis have a predictive value for survival time of 72.6% (*p* < 0.05), with an overall model quality of 0.59. The predictive value of IRIS stage at diagnosis (1 − normal) for survival time was 83.7% (*p* < 0.05), with an overall model quality of 0.73.

### 3.3. The Presence of Coinfections at the Time of Diagnosis Compromised Dog Survival

**Figure 5:**
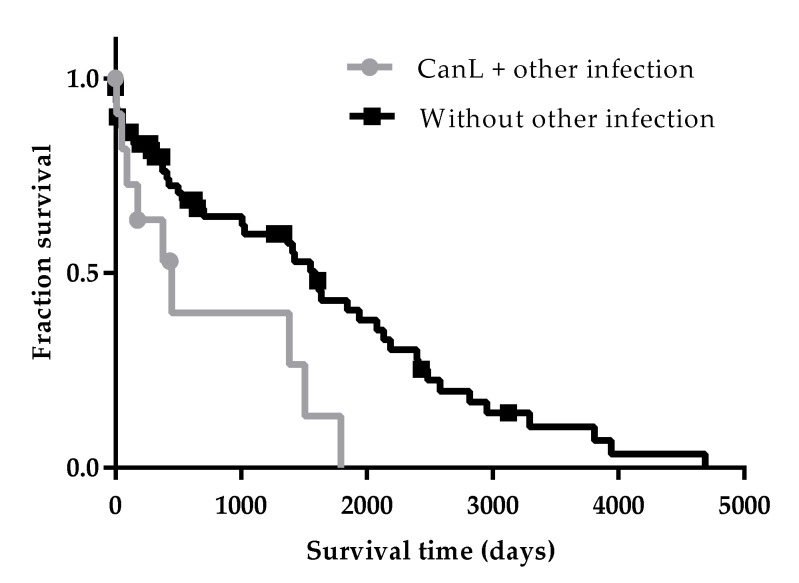
Influence of coinfections at the time of diagnosis on the survival time of patients with CanL. Log-rank (Mantel–Cox) test was used to compare the survival time of patients with CanL and patients with CanL and others infectious diseases. Both events (death) and censored data were considered. Dots and squares represent censored dogs.

Specific tests have been performed in case of suspicion of concomitant infections. Out of 16 dogs tested for hemoparasites (*E. canis*, *B. canis*, and *R. conorii*), nine were positive and received specific treatment. From six dogs tested for *Leptospira* spp., one was positive. One dog tested for *N. caninum* was positive. Both animals received appropriate treatment. Bilateral eye infestation by *T. callipaeda* was identified in one patient and the animal was treated appropriately. Median survival time of dogs with others infectious diseases (448 days) was significantly lower compared with dogs where no other infectious disease was diagnosed (1587 days) (*p* = 0.0142) (Figure 5).

### 3.4. Anti-Leishmania Therapy Can Influence Prognosis

**Figure 6:**
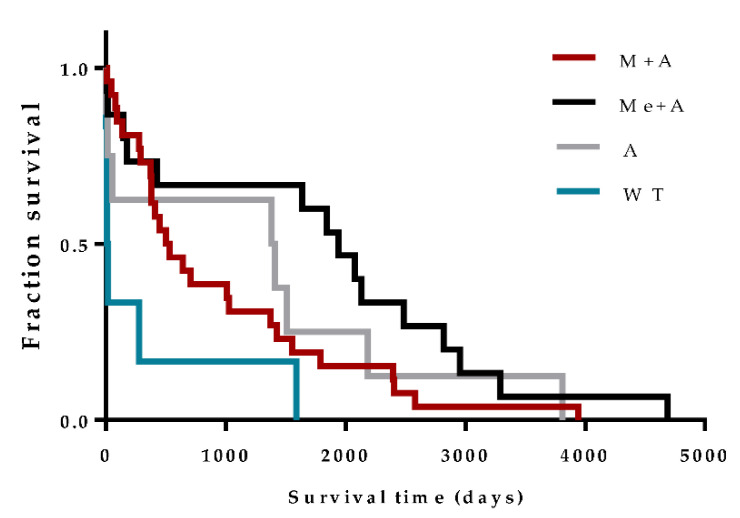
Influence of treatment on the survival time of patients with CanL. Log-rank (Mantel–Cox) test was used to compare the survival time of patients treated with miltefosine in association with allopurinol (M + A), meglumine antimoniate in association with allopurinol (Me + A), allopurinol (A) and untreated dogs (WT). Only events (death) were considered.

Considering only events (deaths), the median survival time of dogs that received Me + A, A and M + A was 1940, 1396 and 514.5 days, respectively, while untreated dogs presented a median survival of 12 days. The median survival time of dogs treated with Me + A was significantly higher when compared with those treated with M + A (*p* = 0.0452) and dogs that did not receive treatment (*p* = 0.0009). Administration of M + A promoted a significant increase in the survival time of dogs when compared with untreated dogs (*p* = 0.0229) (Figure 6). 

**Figure 7:**
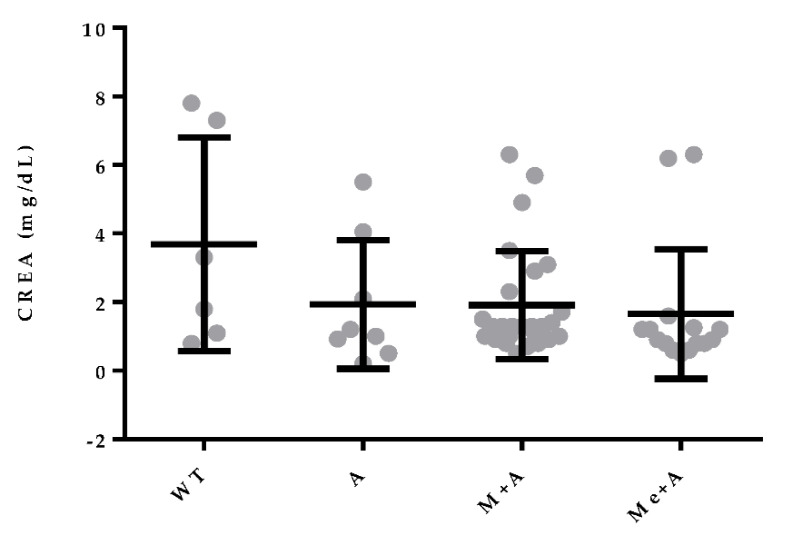
Selection of therapeutic protocol according to blood creatinine levels. Levels of creatinine presented by dogs at the time of CanL diagnosis that were not treated (WT), treated with allopurinol alone (A), miltefosine plus allopurinol (M + A) or meglumine antimoniate plus allopurinol (Me + A) represented by mean and standard deviation. Only events (death) were considered.

Since the selection of therapeutic protocol may have been influenced by renal function, we compared blood creatinine levels of dogs that received different therapeutic protocols. Mean blood creatinine levels of dogs that received A, M + A or Me + A were 1.934 mg/dL, 1.915 mg/dL and 1.657 mg/dL, respectively, although differences were not statistically significant. Untreated animals presented a mean creatinine level of 3.683 mg/dL (Figure 7).

**Figure 8:**
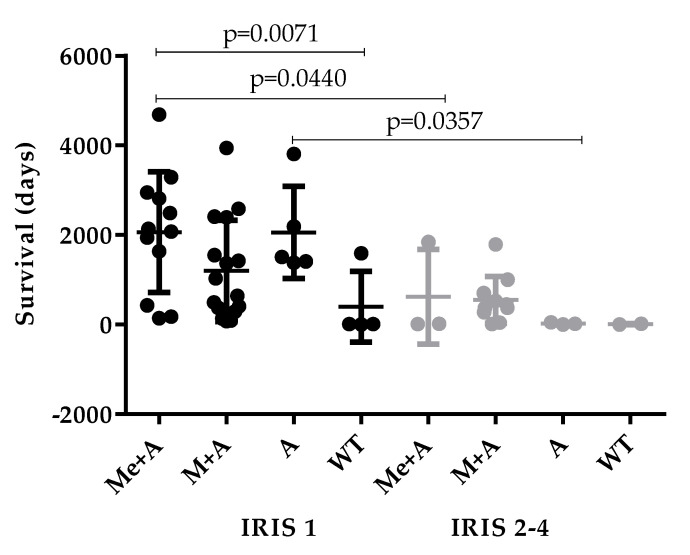
The survival time of patients according to the IRIS stage and therapeutic protocol. The survival time of patients showing normal renal function (classified as IRIS 1) or presenting renal dysfunction (IRIS 2, 3, and 4) treated with meglumine antimoniate plus allopurinol (Me + A), miltefosine plus allopurinol (M + A), allopurinol (A) or that did not receive treatment (WT) are represented by the mean and standard deviation. Statistical analysis was performed using the nonparametric Mann–Whitney test. Statistically significant differences are indicated by capped lines (*p* < 0.05). Only events (death) were considered.

Patients without renal dysfunction (IRIS 1) treated with Me + A (*p* = 0.0440) or A (*p* = 0.0357) presented higher survival time compared with those with renal dysfunction (IRIS 2–4) and submitted to the same therapeutic protocol. Furthermore, a significant increase in survival time was observed in dogs with normal renal function treated with Me + A compared to those untreated (*p* = 0.0071) (Figure 8).

### 3.5. The Improvement in Renal Function Is a Rare Event during the Course of CanL Treatment

Considering only events, the IRIS stage worsened in 25% (14/56) of the dogs during the disease outcome, including eight dogs that were classified as IRIS 1 at the time of diagnosis and that later developed renal dysfunction. Only three of 56 dogs experienced a long-term improvement in renal function (one dog treated with Me + A, one dog treated with M + A and another dog with A in monotherapy). Renal dysfunction (IRIS 3 and 4) was the cause of death or euthanasia in 19 (33.9%) dogs. Death for the remaining animals was associated with non-infectious diseases, such as tumors, cardiac disease, hepatic disease and pyometra that developed during CanL evolution.

Around 53.5% of the censored dogs were lost during follow-up in the first year after diagnosis while the remaining dogs maintained regular veterinary follow-ups.

## 4. Discussion

Estimating prognosis is a fundamental step to make fully informed clinical decisions [21]. However, in patients with CanL, this task is difficult to carry out due to the lack of scientific evidence. Despite some limitations—in particular, its retrospective nature and the lack of some laboratory data that would allow better staging of the patients—this multicenter study, based on statistical analysis of different methodologies, outlines some generalizations on the prognosis of dogs naturally infected by *Leishmania*, living in zoonotic visceral leishmaniosis endemic regions.

CanL is endemic in Portugal, registering seroprevalences between 5.6% and 12% in different districts of the Alentejo region [53,54]. The majority of dogs included in this study were mixed breed and Labrador retriever dogs, probably reflecting the characteristics of the dog population in this region. Although the Labrador breed has no especial predisposition to *Leishmania* infection, some studies report atypical cases of CanL in this dog’s breed [55,56,57]. In domestic dogs, age seems to be an important risk factor, with a bimodal distribution of the disease, the highest prevalence being reported in dogs younger than three years and older than eight years [58,59]. Contrary to these studies, in our work, the disease was mainly diagnosed in adult dogs with ages ranging from four to eight years. The discrepancies observed may be related to the delay in visiting the veterinarian to diagnose the infection, a consequence of the long asymptomatic period of CanL [60]. We found a slightly higher proportion of CanL in males [61,62], which can be justified by the characteristic roaming behavior of male dogs [53]. Furthermore, adult males are often used for guarding and other outside occupations, being more exposed to the vector.

In the vast majority of dogs, the diagnosis of CanL was established based on the results of quantitative indirect serological tests associated with clinical signs and/or clinicopathological abnormalities compatible with *Leishmania* infection. A high level of antibodies is usually associated with high parasite density and confirms the diagnosis of CanL in dogs with clinical or clinicopathological suspicion [3,63]. When clinical suspicion was high but serological tests were found inconclusive (three animals), the diagnosis of CanL was established by the identification of *Leishmania* amastigote (direct diagnosis) in skin and spleen cytology, as is recommended by several authors [3,23].

In twenty dogs, the results of qualitative RIT tests were not confirmed by quantitative serological tests due to economic restrictions. Although RIT based assays are easy to use and provide rapid results, the performance is still not optimal [25,64,65]. Furthermore, in these cases, failure to determine the level of antibodies compromises continued patient management.

In terms of clinicopathological profile, moderate normocytic, normochromic anemia was a common finding, as is frequently reported in the literature [51,66,67]. In our study, the presence or absence of anemia did not influence prognosis, but dogs with mild anemia survived longer than dogs with severe anemia. Anemia has a multifactorial origin related to the inflammatory chronic disease, reduced erythropoietin synthesis due to renal failure, hemorrhage, immune-mediated mechanisms and medullar hypoplasia [16,51,68]. Severe anemia is frequently referred to as a poor prognosis factor in non-infectious and infectious diseases [69,70]. As anemia due to CanL is usually mild to moderate, the presence of severe anemia might raise the suspicion of other coinfections, particularly ehrlichiosis [28,69] and babesiosis [71].

Uremia and creatinemia at the time of diagnosis were not frequent clinicopathological findings. The majority of dogs that were followed retrospectively in this study were classified as IRIS stage 1. Indeed, although renal involvement occurs in virtually all dogs with CanL [18], azotemia is not a common finding [2,67]. Renal disease, caused by glomerular deposition of circulating immune complexes, progresses from asymptomatic proteinuria to nephrotic syndrome and/or CKD [3,19,20,72,73]. CanL patients start to develop proteinuria in the absence of azotemia [72] and becomes evident when advanced stages of the disease azotemia are reached [2,51]. Despite the high proportion of dogs exhibiting normal renal profiles at the time of diagnosis, a considerable number of dogs under treatment progressed to CKD, which constituted a relevant cause of death in our study, as already documented [23].

Although proteinuria is a frequent finding in CanL [3], in our study, its quantification was only performed on a small number of animals, which limited the correct clinical staging of patients with CanL and monitoring of the response to treatment. Dogs’ owners pointed out economic restrictions as the main reason for not carrying out urinalysis and UP/C. 

Renal dysfunction is considered the main negative prognostic factor for dogs with leishmaniosis [3,22]. It was estimated that around 75% of dogs without evidence of renal involvement at diagnosis live for more than four years if adequately treated [23]. Our study reinforces the idea that dogs with normal renal function at the time of CanL diagnosis have a better prognosis. Thus, patients with normal blood urea levels and those classified as IRIS 1 at the time of diagnosis survived considerably longer than dogs classified as IRIS 2, which survived for around two and a half years, and dogs staged as IRIS 3–4, which survived around one month.

CanL patients are often concurrently infected with other pathogens [30,32]. In our study, a small proportion of dogs were tested for other infectious diseases, justifying the low frequency of coinfection compared with other studies [29]. Despite the reduced proportion of dogs positive for other infectious diseases at the time of CanL diagnosis, the survival time of these dogs was significantly lower compared with dogs free of other infectious diseases. The mixed Type 1 T helper (Th1) and Th2 immune response developed by susceptible dogs in response to *L. infantum* infection [1,2,74] may suppress the immune system, promoting the reactivation of a previously subclinical infection or facilitating the establishment of new infections in dogs [28].

The therapeutic protocol can influence CanL prognosis [23]. In the present study, dogs treated with combined therapy (M + A and Me + A) experienced a long survival time compared with dogs that did not receive treatment. In terms of combined therapy, dogs treated with Me + A survived longer than those treated with M + A. However, these results must be interpreted with caution since, in general, veterinarians prescribe M + A [36] or A in monotherapy to dogs evidencing more clinicopathological alterations, and Me + A to animals that show fewer abnormalities [36], in order to minimize renal damage. The selection of pharmacological therapy with a low renal impact, particularly in dogs with previous renal dysfunction, is essential [26,75]. Treatment with M seems to have a limited impact on kidney function [75] and the combined therapy M + A is safe for the management of dogs with leishmaniosis, particularly in those with impaired renal function at the time of diagnosis [26]. Although, in our study, blood creatinine levels of dogs treated with A in monotherapy and M + A were higher compared with the creatinemia of dogs treated with Me + A, the differences were not significant. 

When different therapeutic protocols were evaluated in accordance with IRIS stage, the positive impact of Me + A on dog survival was also evident. Indeed, although without statistically significant differences, M + A seems to promote a shorter survival time when compared to Me + A in dogs classified as IRIS 1. This finding is not surprising, since long-term follow-up of CanL dogs revealed that those treated with Me + A showed more stable clinicopathological findings and low incidence of recurrences compared with those treated with M + A, thus indicating that Me has better clinical efficacy than M [49]. In addition, treatment with A in monotherapy had a positive effect on life expectancy, similar to Me + A in dogs classified as IRIS 1 but not IRIS 2–4. However, these results must be observed carefully, due to the low number of dogs treated with this therapeutic protocol. This finding is unexpected since it was demonstrated that A restores clinical and clinicopathological abnormalities but does not generate a sustained reduction in parasite load [76,77]. 

As seems obvious, in our study, untreated dogs had a shorter survival time and increased levels of blood creatinine levels, probably associated with severe renal dysfunction. Indeed, a recent study confirms that dogs that do not receive treatment experience an increase in infection levels and a progressive decline in their clinical condition [76,77]. The option of not treating or euthanizing these dogs immediately after diagnosis seems to be related to the poor prognosis of severe and very severe disease [3], lack of therapeutic evidence for these stages of CanL and tutors’ economic constraints to support the expensive anti-*Leishmania* treatment.

CanL is a chronic disease that requires constant veterinary monitoring to identify and treat relapses and *Leishmania*-related organ dysfunction, which entails high costs for dogs’ tutors [44]. In this study, around 30.3% of dogs abandoned the clinical follow-ups. Furthermore, clinical records frequently refer to economic constraints to justify the absence of some laboratory analyses and the refusal to administer the prescribed medication suggested by the veterinarian. Lack of anti-*Leishmania* treatment and the abandonment of clinical follow-ups can have serious repercussions in terms of animal and public health, as the uncontrolled disease should have a high impact on parasite transmission.

## 5. Conclusions

In conclusion, this study reinforces the value of blood urea and creatinine levels as prognostic factors in CanL. In addition, severe anemia and the presence of coinfections at the time of CanL diagnosis were correlated with shorter survival, and the combined anti-*Leishmania* therapy Me + A was related to prolonged surviving. It is a matter of concern that a considerable number of dogs did not receive anti-*Leishmania* treatment and abandoned the clinical follow-ups, which may have serious repercussions in terms of animal wellbeing and public health.

## Figures and Tables

**Table 1:** Patient signalment, n = 99.

		Patients (%)
Sex	Male	59.6
	Female	40.4
Breed	Mixed-breed	47.5
	Labrador retriever	16.2
	Boxer	5.0
	Epagneul Breton	4.0
	German Shepherd	3.0
	Other breeds	24.3
Age at diagnosis (years)	Young (<1)	1.0
	Young adult (1–3)	18.2
	Adult (4–8)	53.5
	Mature (> 8)	27.3

**Table 2:** Clinical signs compatible with canine leishmaniosis at the time of diagnosis, n = 95.

Clinical Signs	Patients (%)
Dermatological signs	67.4
Weight loss	40.0
Lymphadenomegaly	21.0
Osteoarticular pathology and muscle atrophy	18.9
Ocular pathology	14.7
Splenomegaly	10.5
Gastrointestinal signs	10.5
Anorexia	10.5
Epistaxis	4.2
Fever	2.1
Others	21.0

**Table 3:** Clinicopathological abnormalities compatible with CanL at the time of diagnosis, n = 99.

Analytic Panel	Abnormalities	Reference Range	Patients (%)
Erythrogram	Anemia	Ht 37–55%	60.4
	Mild anemia	Ht 30–37%	44.8
	Moderate anemia	Ht 20–30%	51.7
	Severe anemia	Ht < 20%	3.4
Leucogram	Leukocytosis	6.0–17.0 (×10^9^/L)	9.6
	Leukopenia	6.0–17.0 (×10^9^/L)	7.4
	Lymphocytosis	0.8–5.1 (×10^9^/L)	9.6
	Lymphopenia	0.8–5.1 (×10^9^/L)	2.1
	Monocytosis	0.0–1.8 (×10^9^/L)	18.0
	Neutrophilia	4.0–12.6 (×10^9^/L)	7.3
	Neutropenia	4.0–12.6 (×10^9^/L)	10.4
Platelet count	Thrombocytopenia	117–460 (×10^9^/μL)	18.3
Biochemical profile	Hyperproteinemia	5.5–7.5 g/dL	52.8
	Hypoproteinemia	5.5–7.5 g/dL	8.8
	Creatinemia	0.4–1.2 mg/dL	18.3
	Uremia	6–24 mg/dL	43.2
	Elevated ALP	0–85 UI/L	12.0
	Elevated GPT	13–92 UI/L	10.4

Ht—hematocrit; ALP—alkaline phosphatase; GPT—glutamic-pyruvate-transaminase; IU—international units.
